# NEWS

**DOI:** 10.7189/jogh.04.010202

**Published:** 2014-06

## THE BILL AND MELINDA GATES FOUNDATION

• Billionaire Warren Buffett donated US$ 2.6 billion of Berkshire Hathaway Class B shares to the Bill and Melinda Gates Foundation, plus four charities linked to his family. The “Oracle of Omaha” is a notable philanthropist who has been giving annually to the foundation since 2006, with an estimated total donation of US$ 1.5 billion in shares. It exceeds Mr. Buffett's last contribution, when he donated US$ 1.52 billion in 2012. He has pledged to give away 99% of his fortune upon his and his wife's demise. By 2012, he inspired 83 fellow billionaires to make similar pledges, agreeing to give away at least half their fortunes during their lifetimes. (*Forbes*, 8 July 2013)

• Russian investor Yuri Milner, a physicist by training, is looking to apply his scientific approach to philanthropy. He is one of seven billionaires to recently sign the Giving Pledge, which commits its current 122 signatories to giving at least half their wealth to charitable organizations. As well as encouraging philanthropy amongst the world’s wealthiest, the Pledge, founded by Warren Buffet and Bill and Melinda Gates, aims to inspire others towards giving and sharing knowledge. “In this season of giving, we are inspired by the millions of people across the global who give what they can in meaningful and significant ways. The Giving Pledge is rooted in this spirit, and hopes to inspire people to tackle problems that are inherently difficult and diverse in an effort to address the pressing social problems they care about most,” says Melinda Gates. (*Forbes,* 10 Dec 2013)

• The BMGF named its new Chief Executive as Susan Desmond–Hellman, the chancellor of the University of California, San Francisco (UCSF), and an oncologist and public health expert. She took up the post on 1 May 2014. Prior to UCSF, she was president of product development at biotech pioneer Genentech, where she led the development of two of the first gene–based cancer drugs, Herceptin and Avastin. (*Reuters,* 17 Dec 2013)

• In an interview with the *Wall Street Journal*, following the Foundation's “2014 Open Letter”, Bill and Melinda Gates spoke about the state of today’s world – better today by nearly all measures. They debunked three myths on the world’s poor. First, poor countries are not doomed to stay poor; more than half the world’s population lives in a new class of middle–income countries, and they predict that there will be almost no poor countries left by 2025. Second, foreign aid investment is a huge success by saving lives and founding long–term economic progress; it is not a waste of money, and potential corruption should not deter donors. Finally, saving lives does not cause over–population; instead falling death rates cause falling birthrates. There is a greater chance of creating a world where extreme poverty is the exception not the rule, if these myths are less widely believed. (*Wall Street Journal*, 17 Jan 2014)

• In a wide–ranging conversation with the American Enterprise Institute, Bill Gates spoke about falling global poverty and child mortality rates, the developmental challenges faced by African countries, the role of government and philanthropy in addressing market failures, the US education system, the Foundation’s priorities, and how to measure the impact of aid. He talked about the importance of the private sector in African agriculture, as farmers above the subsistence level can provide better diets for their children, with more resilience during hardship. He advised on how people can make a difference by focusing on a small number of causes, be engaged with them, and stay with them long–term. He shared his views on the long–term trends in the jobs market, saying that more jobs are likely to be automated, and called for reforms to the tax system by switching taxation from payroll to consumption and capital to increase demand for labour. (*American Enterprise Institute,* 13 Mar 2014)

## THE GAVI ALLIANCE

• The 2013 *Publish What You Fund* index ranks the transparency of donor organisations, by commitment to aid transparency, organisation–level publication of financial information and general plans, and the availability of country–specific project activities; and 2013 saw the format of publishing feature in the rankings. The index is restricted to influential donors which spend over US$ 1 billion a year, and must be committed to transparency. The leaders in aid transparency were the USA’s Millennium Challenge Corporation (strong in publishing information in machine–readable formats), the UK’s Department for International Development (where transparency is part of its agenda), and GAVI Alliance (the only agency to publish all information in a machine–readable format). (*Devex*, 24 Oct 2013)

• The US has committed an additional US$ 175 million to funding GAVI vaccination programs; a record amount for the US government and comes as GAVI prepares for its 2016–20 funding replenishment. GAVI has undertaken an unprecedented acceleration of its programs, from 55 rollouts in 2011–12 to more than 150 in the following years, including the inactivated polio virus. Ensuring that the benefits of vaccination reach every child is an important focus of the next cycle. (*GAVI*, 17 Jan 2014)

• At the World Economic Forum at Davos, GAVI announced a US$ 152 million initiative to immunise children against disease using private sector partnerships, which leverages cash and expertise from corporations and foundations. These partnerships have a critical role in improving global health, and there were calls for increased participation from the private sector to reach the 22 million children who go unvaccinated annually. “Every 20 seconds, a child dies from preventable diseases,” said Ms Justine Greening, Britain’s international development secretary. (*GAVI*, 28 Jan 2014)

• GAVI announced its support of vaccination programmes in Rwanda, Uganda and Uzbekistan, to protect 1.5 million girls against the causes of cervical cancer. The first national roll–outs will begin in Uganda and Uzbekistan in 2015, while Rwanda moves from a vaccine manufacturer’s donation to GAVI support to secure its existing programme. Cervical cancer was described by GAVI as a “scourge” on women and their families in developing countries, where limited screening and treatment make vaccination a vital prevention tool. These countries have developed detailed plans to ensure that girls aged 10–12 years are vaccinated in schools, and that girls not in education are included reached via community outreach. (*GAVI*, 8 Mar 2014)

• Rotavirus vaccines have been introduced into Cameroon’s routine immunisation programme, with support from the GAVI Alliance. This life–saving virus could have a massive impact on children’s health, as each year rotavirus claims almost 6000 lives and causes an estimated one–third of all diarrhoeal hospitalisations in Cameroon in children aged under five. Cameroon would have a greater chance of reaching the Millennium Development Goal of reducing child mortality if every child is fully vaccinated with the recommended vaccines before their first birthday. “Rotavirus vaccine is a very effective way of protecting children against rotavirus diarhoeal disease and can be used in addition to other general diarrhoea prevention methods including hand washing with soap, drinking potable water and using latrines,” said Dr Charlotte Faty Ndiaye, WHO representative in Cameroon. (*GAVI*, 28 Mar 2014)

## THE WORLD BANK

• A coalition of developed and developing countries pledged to accelerate efforts to end extreme poverty, by committing a record US$ 52 billion to the World Bank’s International Development Association. There will be more emphasis on challenging frontier areas and private sector mobilisation, and investment in gender equality and climate change is key to the future; all is underpinned by a commitment to equitable growth. Amongst others, the support will provide eg, electricity for 15–20 million, vaccines for 200 million children and extend basic health care and clean water supplies. It will run from 2014 to 2017, thus spanning the MDG and post–2015 agenda. (*World Bank,* 17 Dec 2013)

• Cambodia has exceeded the Millennium Development Goal’s poverty target, and is one of the best global performers in poverty reduction. The share of people living in absolute poverty (US$ 1.15/d) fell from 53% to 20.5% between 2004 and 2011. The main drivers were increased rice production and prices, with resulting higher revenue and wages. However, most people still live too close to the poverty line, and decreasing numbers of people in absolute poverty has led to a sharp rise of “near–poor” people, from 4.6 million in 2004 to 8.1 million in 2011. Small income shocks (eg, a loss of US$ 0.30/d) could throw 3 million back into poverty. Cambodia’s development goals focus on helping the remaining 20% escape absolute poverty, and prevent the near–poor from slipping back, emphasizing growth and equity. The Bank recommends infrastructure improvements, broadening access to education and health services, and reducing child malnutrition as key to lifting more people out of poverty. Crop diversification and enhanced rice profitability will help prevent the near–poor slipping back into absolute poverty. (*World Bank*, 19 Feb 2014)

• The World Bank has delayed approving a US$ 90 million loan to support Uganda’s health system. The Bank’s President, Mr Jim Kim, said that the Bank is reviewing wherever recent changes in Ugandan law would lead to discrimination against gay people in the maternal and family planning projects it supported. He stressed that it will continue to help fight poverty in Uganda. The Bank has previously used this tactic to express concerns about human rights, eg, freezing loans to China after Tiananmen Square. “Anti–discrimination and equality might be part of our moral values as individuals, but for us the even more important thing is now we’ve got a lot of good data that suggests it’s bad for economic growth too,” says Mr Kim. (*Devex*, 14 Mar 2014)

• Tackling the pervasive inequality faced by women farmers in Africa is vital to tackling poverty, increasing economic growth and feeding its population. A World Bank report examined the scale and differences between male and female farmers in six African countries, identifying gender gaps, factors holding back female farmers, and actions to reduce inequality. It found that female farm productivity is 13–25% lower than male in the countries surveyed. It estimates that given equal access to resources, women farmers could increase farm yields by 20–30%, thereby improving food security, economic growth and creating job opportunities for millions of young Africans. The report calls for governmental action to close the gender gap, eg, by improving female land rights, use of agricultural techniques, education, and market and childcare access. (*World Bank*, 18 Mar 2014)

• The World Bank, The Gates Foundation and other donors are increasing efforts against neglected tropical diseases in low–income countries with a US$ 240 million injection of new funding. This follows a pledge two years ago by several pharmaceutical companies (Sanofi, GlaxoSmithKline, Merck etc.) to donate medicines to tackle parasitic and bacterial infections that threaten 1–in–6 people worldwide. Half of the funding will be spent on combating soil–transmitted helminthes – intestinal worms that commonly affect children living in poverty. The World Bank Group has also committed US$ 120 million to support the fight against neglected diseases, including school–based worming programmes. (*Reuters*, 2 Apr 2014)

## UNITED NATIONS

• *The Lancet* published reflections on the UN General Assembly on delegates’ wish to focus on MDG successes, rather than on learning from missed opportunities. Three lessons already apparent from the MDG agenda were outlined. First, MDGs have created a vast aid industry that can cause duplication and confusion without a sustainable legacy. Second, unacceptable inequities in health will persist and worsen unless a proper financing facility is created. Lastly, short–termism has incentivized interventions that can be deployed and measured quickly, whilst overlooking the need for skilled health workers, accurate information and quality care in health care. (*The Lancet*, 5 Oct 2013)

• At its 68th session, the UN General Assembly adopted a US$ 5.53 billion budget to finance its activities over the next two years, including its global judicial, humanitarian and peace–keeping operations. It also adopted more than 20 texts on a range of issues, eg, financing international criminal tribunals. Of particular note was the State of Palestine casting its first ballot, one Member State declining a seat on the Security Council, and remembrances of Nelson Mandela highlighting the world’s need for peacekeepers. The post–2015 development agenda is the proposed theme for the general debate, with several high–level thematic debates scheduled for 2014, to be concluded with a stock–taking event in September. (*UN News,* 27 Dec 2013)

• Research by the University of Kent shows that more than 70% of religious NGOs at the UN are Christian, and the Vatican has special observer status as both a state and religion. Islam is represented via a collection of states rather than civil society NGOs, and Asian religions (eg, Buddhism, Hinduism) are unrepresented, with funding being a major barrier to access. It found that the overall number of inter–faith and new–age NGOs is small but they can have a disproportionate influence, having many meetings with UN diplomats. It calls for more awareness, transparency and equality in how religious NGOs work in the UN, and more understanding of how religions enhance and constrain human rights. (*The Guardian,* 1 Jan 2014)

• The UN has released early results from its far–reaching global survey MyWorld2015. The ongoing survey asks people which factors would improve their and their families’ lives. Results will be shared with the Secretary General and others leading up to the post–2015 development agenda. Early results suggest that education is a main priority for two–thirds of respondents, and health is a priority for those aged over 61. Otherwise, priorities tend to be specific to circumstances and countries. However, climate change action scores poorly. Although this may be caused by the question’s wording and could change, it is concerning to those hoping to combat climate change by individual action, as it seems many are not interested. (*The Washington Post,* 20 Feb 2014)

• The UN faces a lawsuit over the cholera outbreak in Haiti that is blamed on its peacekeepers. The UN does not accept responsibility for the outbreak, which has killed more than 9000 people. The lawsuit alleges that the outbreak arose from “negligent, reckless, and tortious conduct”, beginning after the arrival of UN troops after the 2010 earthquake who were not screened for cholera. The UN hired a private contractor to ensure sanitary conditions, however contaminated sewage leaked into water supplies, resulting in Haiti’s first cholera outbreak for 150 years. The UN claimed immunity, but the lawsuit argues that the UN is not immune from liability in such cases. (*The Guardian*, 12 Mar 2014)

## UN AIDS and The Global Fund

• The WHO says that millions of young people are at risk of HIV infection due to inadequate health services, as AIDS–related deaths increased by 50% amongst 10–19 years old from 2002 to 2012, despite falling by 30% amongst the general population. This is ascribed to government failures in prioritising adolescents in national HIV plans, lack of teen–friendly testing services and counseling, plus inadequate treatment of people who are born with HIV. WHO have issued new guidelines on HIV support and care for adolescents, calling for more tailored services, immediate treatment, and support for status disclosure and treatment compliance. WHO calls for action to tackle barriers to HIV treatment and prevention, and other groups call for a “prevention revolution,” particularly amongst young people and marginalised groups, to accelerate the global fight against HIV and AIDS. (*The Guardian,* 26 Nov 2013)

• The world’s donor countries pledged US$ 12 billion over three years to the Global Fund to Fight AIDS, Tuberculosis and Malaria. Although an increase over the 2010 pledge, it falls short of the hoped–for US$ 15 billion. US$ 15 billion would help 85% of people in need; US$ 12 billion would reach 68%. The goal of ensuring that the increasing numbers who test positive for HIV receive treatment will not be met at this donation level, the ongoing battle against malaria needs more investment if malaria deaths are to hold steady yet alone further fall, and tuberculosis is rising in line with the number of HIV cases because people with compromised immune systems are more vulnerable to it. Reactions on the pledge were divided; some advocates are pleased that donations have increased, whilst others call for rich countries to contribute more. (*New York Times*, 3 Dec 2013)

• An outsider could perceive that the UN’s global health agencies are confused and complex, argues Jon Lidén, Centre on Global Health Security, and other agencies, such as NGOs and Foundations, are perceived as more responsive and innovative. Against this background, UN agencies are discussing major structural reforms. However, much of the progress in global health in the past decade could not have happened without the UN agencies, and for some areas (eg, accident and violence prevention etc), there are no alternatives. He argues that, aside from UNAIDS’s Michel Sidibé, the UN lacks new ideas, vision and leadership. The UN is most effective when its leaders set bold agendas for others to follow, rather than second–guessing member states’ priorities and worrying about other agencies’ activities. (*Chatham House,* 18 Dec 2013)

• Bangladesh has a history of endemic malaria transmission in many districts. The Global Fund gave funding to the Bangladesh National Malaria Control Programme, and there was a follow–up epidemiological and economic assessment of the country’s malaria control. It uncovered a general reduction in malaria cases, mainly due to the widespread use of nets, more use of rapid diagnostic tests and antimalarial treatments, and a high number of health workers and facilities. Insecticide–treated nets were cheaper, making the preventative measures highly cost–effective. Bangladesh is now moving from control to elimination in some districts, making total elimination an achievable prospect. However, consistent funding is essential to avoid the inevitable resurgence if control and surveillance efforts are scaled back. (*The Lancet Global Health,* 1 Feb 2014)

• Studies show that intimate partner violence can increase the risk of HIV infection by 50%, and that one–in–three women experience intimate partner violence at some point. Violence, or fear of violence, can undermine access to treatment, care and support services for women living with HIV. UNAIDS has called for governments and communities to take action against violence against women, arguing that it is not only a human rights violation, but also makes them more vulnerable to HIV infection. Women living with HIV are more likely to be subjected to violence, women most vulnerable to violence are also most vulnerable to HIV, and violence undermines the HIV response by creating a barrier to accessing services. (*UNAIDS*, 12 Mar 2014)

## UNICEF

• The EU announced a US$ 431 million allocation to UNICEF to improve maternal and child health and nutrition in 15 developing countries. It will focus on under–nutrition and infectious diseases – among the root causes of child mortality – and other programmes will focus on improving access to water, sanitation, medical services and nutrition. This builds on achievements towards the MDGs, and accelerates progress towards MDG4 – reducing mortality in children under 5 years by two–thirds – which otherwise will not be reached until 2028. UNICEF and the EU, in partnership with governments and other agencies, will scale–up interventions to reduce child mortality and improve maternal and pre–natal health. (*UNICEF*, 4 Feb 2014)

• UNICEF’s report, *Every Child’s Birth Right: Inequities and Trends in Birth Registration,* found that in 2012 only 60% of newborns were registered, equating to unregistered 230 million children aged under 5 world–wide. These children are at risk of being unable to access government programmes such as education and health care, are more vulnerable to neglect and abuse, and in the longer–term it may affect their citizenship and right to vote. Children in certain groups or in impoverished or remote areas are less likely to be registered. UNICEF is developing low–cost technology to identify and report unregistered births, working in countries to register newborns and bring them into existence in the eyes of government. (*TIME*, 10 Dec 2013)

• UNICEF appealed for US$ 2.2 billion to provide essential humanitarian aid in 2014 to 85 million people, including 59 million children, who face conflict, natural disasters and other emergencies across 50 countries. UNICEF said that the children and families displaced by conflict in South Sudan join millions more affected by conflict in the Central African Republic and Syria. However, many other desperate situations with less media focus need immediate funding and urgent aid, eg, Afghanistan, Colombia, the Democratic Republic of the Congo, Myanmar, Somalia and Yemen. For Syria and the sub–region, UNICEF is appealing for US$ 835 million to deliver life–saving assistance including immunisation, water, sanitation, education and protection, and to investment in sustainable futures. UNICEF is also seeking funds that are not earmarked for specific projects, to enable it to respond to underfunded emergencies or where needs are greatest. *(UNICEF*, 21 Feb 2014)

• The Philippines is gradually recovering from the Nov 2013 Typhoon Haiyan, shown by health centres re–opening, improved supplies of clean water, and children returning to school. Yet children’s needs remain great, and visible destruction is a reminder that much needs to be done to restore devastated lives and communities. Despite intense relief efforts with significant achievements, recovery is likely to be protracted. UNICEF and its partners are focusing on efforts to improve community resilience, providing urgently needed humanitarian assistance and restoring essential services. It has also strengthened its monitoring and information systems on supply inputs, distribution, results and quality. (*UNICEF,* 7 Mar 2014)

• UNICEF is distributing 150 000 mosquito nets and accompanying educational materials, to 75 000 displaced people in the Central African Republic ahead of the impending rainy season, which brings the threat of malaria – particularly deadly to vulnerable populations. Before the crisis, only 36% of the country’s children slept under a mosquito net, and children in displacement sites in makeshift dwellings are particularly at risk from malaria. Since the start of the conflict, malaria has caused an estimated 40% of all illnesses in children aged under 5 years, and sleeping under a mosquito net reduces malaria deaths by 20%. UNICEF partnered with the National Red Cross for the distribution, overseen by the Ministry of Health. (*UNICEF,* 14 Mar 2014)

## WORLD HEALTH ORGANIZATION

• Since 2000, control measures have prevented 3.3 million deaths from malaria, cutting its death rate by 45% (50% in children under five), mainly within the 10 countries with the highest malaria burden. This is a huge pay–off for malaria control and prevention measures. However, WHO stated that absolute numbers are not reducing as quickly as they could. Funding cuts could hinder progress, although agencies recently announced the provision of over 200 million nets over the next 12–18 months. (*Reuters,* 11 Dec 2013)

• The WHO’s *World Cancer Report* reveals the alarming rise in the global cancer burden and the urgent need for effective prevention strategies. There was an estimated 14 million new cases in 2012, which is expected to rise to 22 million by 2022. Cancer deaths are expected to increase from 8.2 million annually to 13 million. Developing countries are disproportionately affected, with 60% of cases and 70% of deaths. Access to effective and affordable treatments would reduce mortality, but is a huge strain on health care systems as the annual economic cost of cancer is an estimated US$ 1.16 trillion. However, vaccination against hepatitis B and HPV, reducing tobacco usage and promoting physical activity and obesity reduction can markedly reduce cancers linked to infections and lifestyle. Low–tech screening and early detection can be highly cost–effective interventions, and the WHO calls for governments of developing countries to enforce regulatory measures and implement cancer prevention plans. (*WHO*, 3 Feb 2014)

• New mortality estimates show that annual measles deaths have reached new lows, dropping 78% from more than 562 000 in 2000 to 122 000 in 2012, with an estimated 13.8 million lives saved by vaccination. Global measles immunisation coverage is a stable 84%, and the Measles and Rubella Initiative has supported mass vaccination campaigns. However, progress towards measles elimination is uneven, with some populations still unprotected and measles is still a global threat. Routine measles vaccination coverage is important in reaching the MDG for child mortality because of its potential to reduce child mortality. Without improved immunisation coverage, outbreaks will continue. The ability to contain outbreaks by improving coverage and, when necessary, implementing high quality vaccination campaigns, requires countries to place a high priority on elimination goals and to invest heavily in health systems improvements. (*WHO*, 6 Feb 2014)

• The WHO’s global action plan set a target for a 25% reduction in non–communicable diseases (NCDs) by 2025; the “25×25” strategy. The plan lists nine voluntary national targets –reducing mortality from NCDs, halting the rise of diabetes and obesity, and other on reduced alcohol intake, smoking and salt, plus more exercise, better blood pressure control and improved treatment. An analysis of the strategy (published in *The Lancet*) calls for NCDs to be measured by mortality and morbidity, thus broadening the range of conditions identified in the strategy (cardiovascular disease, diabetes, cancer and chronic respiratory disease) to include eg, neurological and musculoskeletal diseases. This more comprehensive approach recognises that NCDs can be caused by factors outside individuals’ control (eg, air pollution, environmental and time constraints due to poverty that don’t allow for exercise), so tackling them means action at both societal and individual levels. (*The Lancet Global Health*, 3 Mar 2014)

• According to the WHO, air pollution kills 7 million people globally each year. Fumes from indoor stoves cause half of all deaths – with women and children having higher exposure, as they spend more time indoors – according to the WHO. It causes 1–in–8 deaths and is the biggest environmental health risk. One of its main risks is tiny particles can get deep into the lungs, causing irritation and possibly heart problems. WHO has classified air pollution as a carcinogen, linked to lung and bladder cancers. Experts call for more research on the most dangerous components of air pollution to more effectively target control measures, and individuals can limit their exposure by avoiding travelling at rush hour and taking quieter roads; however there is little evidence that face masks provide protection. (*Associated Press*, 25 Mar 2014)

